# New Method of Preparing Corrosive Sublimate (Hyperoxydated Muriate of Mercury) in the Humid Way

**Published:** 1804-04-01

**Authors:** M. L. Von Schmidt Phiseldeck


### New Method *of preparing* corrosive Sublimate *(hyperoxydated Muriate of Mercury) in the* humid Way. *By* M. L. Von Schmidt Phiseldeck.

It is well known how much apothecaries, desirous of preparing their own medicines, are indebted to Mr. Westrumb, for having furnished them with a method of preparing corrosive sublimate without being exposed to the dangerous vapour it emits during the sublimation. For some time past I have employed myself, merely from scientific views, in preparing corrosive sublimate according to this method. But however much I may be sensible of the advantages of this process, I cannot help regretting the loss sustained in the nitric and muriatic acids, which in general cost so much trouble and expense before they can be obtained pure. I reflected a long time on the means of avoiding this loss, and at length discovered a process much more economical than that of the chemist Hameln. The question was, to dissolve the mercury in the cheapest concentrated acid (this acid, without doubt, was the sulphuric acid,) and to present to the oxyd of mercury the muriatic acid without having separated it from its alkaline base. I resolved then to prepare a solution of mercury in sulphuric acid, and to decompose the sulphate of mercury by muriate of soda. I then hoped hoped that I could easily separate the two salts that were formed by crystallization, as the sulphate of soda for its solution took only eight parts of cold water, whereas corrosive sublimate takes 162; but I found that after the first crystallization the two salts mixed, and that no other means of separating them remained but by alcohol. I shall pass over in silence, the operations which were attended with more or less success in this point of view, and describe only the process which I definitively adopted.

I introduced into a tubulated retort two ounces of mercury and three ounces three gross of concentrated sulphuric acid; I then adapted to the retort a receiver, without luting it, and made a pretty strong fire. During the solution there was disengaged a very considerable quantity of sulphureous gas. When nothing remained in the retort but a white mass, I added a solution of five ounces and a half of marine salt in six ounces of water, and exposed the mixture to strong ebullition for half an hour. A complete solution took place. I filtred the liquid while it was in a state of ebullition, put it into a retort, and distilled it to dryness. On the remaining mass I poured sixteen ounces of alcohol, and caused it to digest for some hours. I then decanted the liquid from off the residuum, filtrated it again warm, and put it once more into the retort after I had washed it, taking care not to spread any of it in the neck of the retort, and distilled it to dryness. I must here remark, that the distilled liquid, which at first had the colour of Malaga wine, assumed, after the solution was concentrated, the colour of water de Rabel; and the saline mass, after the complete evaporation of the alcohol, was exceedingly white. Lime water made no change in the colour of this liquid.

I poured over the mass in the retort twelve ounces of water; I boiled it to solution, and, having filtred the liquor, exposed it to crystallize. Very beautiful crystals in the form of elongated prisms were deposited. I then poured over the residuum eight ounces of new spirit of wine, and again obtained a cosiderable quantity of corrosive sublimate. The distilled liquid, after being rectified on half an ounce of potash, was perfectly pure.

By employing this method, corrosive sublimate, in my opinion, will cost one-half less than by Westrumb's process. Sulphuric acid costs only one-third of what the nitric acid does; and there is no comparison between the price of pure muriatic acid and that of marine salt. I therefore flatter myself that this method will meet with a favourable reception.

